# Dissolved and particulate trace metal micronutrients under the McMurdo Sound seasonal sea ice: basal sea ice communities as a capacitor for iron

**DOI:** 10.3389/fchem.2013.00025

**Published:** 2013-10-30

**Authors:** Abigail E. Noble, Dawn M. Moran, Andrew E. Allen, Mak A. Saito

**Affiliations:** ^1^Stanley Watson Biogeochemistry Laboratory, Marine Chemistry and Geochemistry Department, Woods Hole Oceanographic InstitutionWoods Hole, MA, USA; ^2^Department of Earth Atmospheric and Planetary Science, Massachusetts Institute of TechnologyCambridge, MA, USA; ^3^Microbial and Environmental Genomics Group, J. Craig Venter InstituteSan Diego, CA, USA; ^4^Integrative Oceanography Division, Scripps Institution of Oceanography, University of CaliforniaSan Diego, CA, USA

**Keywords:** Ross Sea, trace metals, cobalt, iron, manganese, sea ice, Antarctica, biogeochemical cycling

## Abstract

Dissolved and particulate metal concentrations are reported from three sites beneath and at the base of the McMurdo Sound seasonal sea ice in the Ross Sea of Antarctica. This dataset provided insight into Co and Mn biogeochemistry, supporting a previous hypothesis for water column mixing occurring faster than scavenging. Three observations support this: first, Mn-containing particles with Mn/Al ratios in excess of the sediment were present in the water column, implying the presence of bacterial Mn-oxidation processes. Second, dissolved and labile Co were uniform with depth beneath the sea ice after the winter season. Third, dissolved Co:PO^3−^_4_ ratios were consistent with previously observed Ross Sea stoichiometry, implying that over-winter scavenging was slow relative to mixing. Abundant dissolved Fe and Mn were consistent with a winter reserve concept, and particulate Al, Fe, Mn, and Co covaried, implying that these metals behaved similarly. Elevated particulate metals were observed in proximity to the nearby Islands, with particulate Fe/Al ratios similar to that of nearby sediment, consistent with a sediment resuspension source. Dissolved and particulate metals were elevated at the shallowest depths (particularly Fe) with elevated particulate P/Al and Fe/Al ratios in excess of sediments, demonstrating a sea ice biomass source. The sea ice biomass was extremely dense (chl a >9500 μg/L) and contained high abundances of particulate metals with elevated metal/Al ratios. A hypothesis for seasonal accumulation of bioactive metals at the base of the McMurdo Sound sea ice by the basal algal community is presented, analogous to a capacitor that accumulates iron during the spring and early summer. The release and transport of particulate metals accumulated at the base of the sea ice by sloughing is discussed as a potentially important mechanism in providing iron nutrition during polynya phytoplankton bloom formation and could be examined in future oceanographic expeditions.

## Introduction

**Figure 1:**
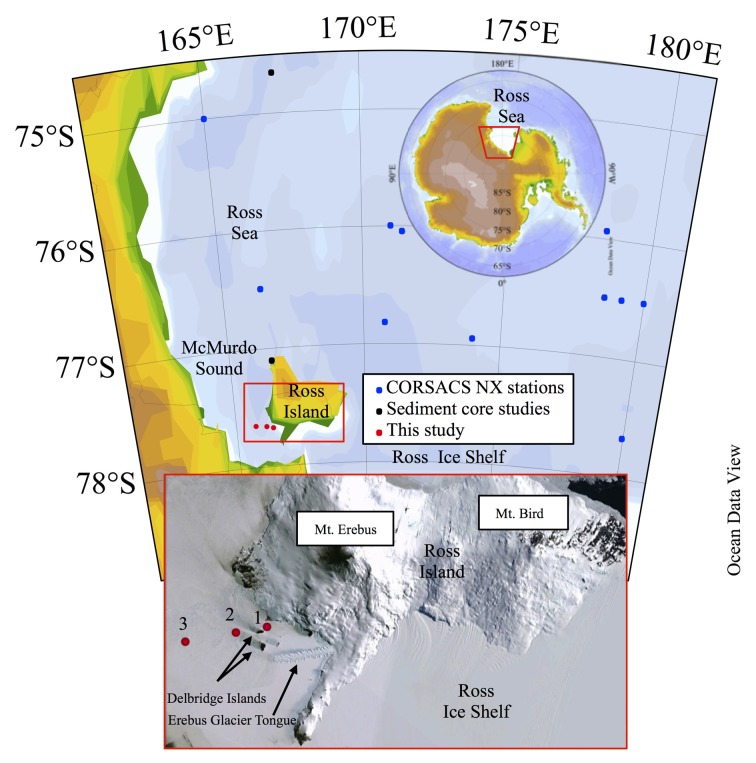
**Maps and satellite photo of the study area.** The red boxes highlight the study area, zooming in from the continent (upper red trapezoid), to the Ross Sea (middle red box), to McMurdo Sound (bottom image) which shows the sampling sites in red circles relative to Ross Island and the Delbridge Islands. Maps were generated in Ocean Data View (Schlitzer, 2011).

The HNLC region of the Ross Sea is an important sink for atmospheric CO_2_, with high pulses of primary productivity and subsequent export occurring during the spring and summer blooms (Arrigo et al., 2008). The Ross Sea also influences the chemical composition of the deep ocean when these waters sink and contribute to Antarctic Bottom Water formation (Orsi and Weiderwohl, 2009). It is a relatively well-mixed shallow sea with an average 500 m depth and gyre-like water movement that drives across the shelf (Smith et al., 2007; Orsi and Weiderwohl, 2009). Though generally covered in ice throughout the winter months, a polynya forms in the springtime and connects it to McMurdo Sound. McMurdo Sound is characterized by an annual sea ice sheet that forms in the winter, melts in the late spring/summer, and feeds into the Ross Sea. Springtime hydrography data suggest that the shallow coastal waters there are well-mixed down to the bottom during the winter (Dinniman et al., 2003; Smith et al., 2007; Figure 1).

A number of factors are thought to impact biological productivity in these waters including light, mixed layer depth, seeding from sea ice, and iron (Leventer and Dunbar, 1996; Smith et al., 1996; Sedwick and Ditullio, 1997; Arrigo et al., 1999; Sedwick et al., 2000). Sedwick et al. (2000) suggested that the availability of iron exerts one of the major controls on phytoplankton growth in the Ross Sea. While iron concentrations have been observed to vary dramatically both seasonally and annually, dissolved iron concentrations are often very low during summer blooms (Sedwick et al., 2000), and can be quickly depleted during the spring bloom as well (Sedwick et al., 2011). This can result in iron limitation of the spring bloom community (Bertrand et al., 2011), and together, these observations raise questions about temporal variation in iron sources required to support phytoplankton blooms in the later months of the summer. Recent studies by Sedwick et al. (2011) and Bertrand et al. (2011) also update the previous assertion that the spring community does not experience iron limitation due to iron input from retreating sea ice (Sedwick et al., 2000).

Evidence of particulate iron and manganese in the Ross Sea suggests that sediment resuspension can be a significant source of these elements to the water column (Corami et al., 2005) and to the sea ice (Lannuzel et al., 2011). Sea ice melt water has also been observed to contribute to particulate iron in surface waters near the receding ice edge during the austral summer (Fitzwater et al., 2000), though some studies suggest that this source is not significant (Lannuzel et al., 2011). In this coastal sea, terrestrial material may influence metal distributions more so than aeolian influences, such as has been observed in Windless Bight on the southern side of Ross Island (Dunbar et al., 2009) and as has been suggested by recent pack and fast ice studies (Lannuzel et al., 2011). Other work suggests that while aeolian input may be small, it is not negligible in Southern McMurdo Sound (Atkins and Dunbar 2009). Regardless of the initial source, elevated concentrations of metals may accumulate in the water column with deep convective mixing during the winter, potentially mobilizing sediment when deep water intrudes onto the shelf. This “winter reserve” has been implicated as a likely source of micronutrients for phytoplankton in the spring, but sampling early season waters during traditional shipboard fieldwork can be difficult (Aguilar-Islas et al., 2008; Sedwick et al., 2011). Sampling the remnants of winter coastal waters was a goal of this field study to test the winter reserve hypothesis.

The distributions of iron, and other important micronutrients such as cobalt and manganese, have all been observed to display nutrient-like profiles in the Ross Sea (Fitzwater et al., 2000; Sedwick et al., 2000; Saito et al., 2010). This is perhaps surprising because cobalt, iron, and manganese are hybrid-type metals, having biogeochemical cycles influenced by redox processes, biological processes, and abiotic processes (Bruland and Lohan, 2004; Noble et al., 2008). The particle reactive characteristic of these metals tends to impose a decreasing concentration trend on their dissolved distributions below the upper water column uptake and remineralization signals (Landing and Bruland, 1987; Martin et al., 1989; Bruland and Lohan, 2004; Saito et al., 2004; Noble et al., 2008, 2012; Shelley et al., 2012). For Mn and Co, scavenging processes have been attributed to the activity of a unique multicopper oxidase enzyme system produced by manganese oxidizing bacteria in the water column (Tebo et al., 1984; Moffett and Ho, 1996; Tebo et al., 2005). We have previously suggested that the absence of a water column scavenging signal in the Ross Sea may be due to mixing on the shelf at a rate that is faster than removal by scavenging and/or alternatively that there is insufficient water column co-oxidation of cobalt by manganese oxidizing bacteria to impose a discernible scavenging signal on the dissolved cobalt vertical structure (Saito et al., 2010).

This study addresses these previous hypotheses through a coordinated dissolved, particulate, and basal sea ice sampling scheme. The data from this study reflect the early season shelf water composition beneath the seasonal sea ice, and aims to examine the potential influence of coastal sources through a transect from McMurdo sound to the Delbridge and Ross Islands. Together, these data provide a useful temporal and geographic study of sources and processes influencing micronutrients in the Ross Sea region.

## Methods

Fieldwork took place on the seasonal sea ice of McMurdo Sound (Expedition Leaders Mak Saito and Andrew E. Allen). Three water column sampling stations were occupied from a mobile sea ice camp between November 9 and 23, 2009. A towed trailer sled (the Thundersled from the previous Andrill program) was modified to include plywood bench space, which was further outfitted as a cleanroom using plastic sheeting and HEPA filter units. The McMurdo Sound station locations were: Station 1 (166.424°E, 77.650°S), Station 2 (166.168°E, 77.659°S), and Station 3 (165.750°E, 77.673°S; Figure 1). All stations were sampled to near bottom with the exception of Station 3, which was sampled to 600 m and exhausted the maximum length of the sampling line. The locations of Stations 1, 2, and 3 are located to the west of Mt. Erebus and north of the Erebus Glacier Ice Tongue (Figure 1), and pass between two of the Delbridge Islands (Tent Island and Inaccessible Island).

### Trace metal sampling techniques

To collect water samples beneath the sea ice, holes were drilled by an ice auger with a 10″ diameter bit and a Jiffy powerhead, and further melted out by a Hotsy heat source at each station. An aluminum tripod was installed over the drill hole and a trace metal clean metering block was suspended above each station's sampling hole to determine sampling depths. A generator-powered winch was used to lower the 8 L X-Niskin sampling bottles (Ocean Test Equipment, Inc., Fort Lauderdale, FL) on a Kevlar line through the sampling hole, and the sampling holes were drilled upwind of the generator to avoid contamination by the fumes. The bottles were tripped using teflon messengers, and then removed from the line upon retrieval and transferred to a trace metal clean lab inside the modified towed trailer sled described above. The bottles were racked under a positive pressure Class-100 hood and were immediately pressurized with 99.999% N_2_ at ~5 psi. Seawater was either collected unfiltered for total dissolvable metals, or filtered through teflon tubing and a 142 mm, 0.4 μm polycarbonate filter within a plastic sandwich filter holder (Geotech Environmental Equipment Inc.). All tubing and filters were acid-washed prior to use. Samples intended for iron and manganese analyses were acidified to pH 1.7 with high purity HCl (Seastar Inc.) within 4 months of sampling, and stored acidified at room temperature for at least 3 months prior to analysis. Samples intended for all cobalt analyses were filled to the top of the bottle and were kept unacidified, at 4°C in darkness until analysis. Previous work has shown good reproducibility between immediate analyses, and reanalyses >17 months after sampling of seawater collected in the Ross Sea, suggesting that, at least for these waters, this preservation and timescale of analyses is more than sufficient (Noble et al., 2012).

A separate filter was used for each X-Niskin bottle sampled. The filters were collected with trace metal clean plastic forceps into cleaned polyethylene bottles and frozen for later particulate analyses.

Low-density polyethylene sample storage bottles were soaked overnight in the acidic detergent, Citranox, rinsed thoroughly with Milli-Q water (Millipore), filled with 10% trace metal grade HCl to soak for 10 days, rinsed thoroughly with Milli-Q water adjusted to pH 2, and double-bagged until use.

### Dissolved cobalt analyses

Concentrations of total dissolved and labile cobalt were determined in the lab 2 months after sampling, using a previously described adsorptive cathodic stripping voltammetry method (Saito and Moffett, 2001; Saito et al., 2004). Measurements were made using the Eco-Chemie μAutolabIII systems connected to Metrohm 663 VA Stands equipped with hanging mercury drop electrodes and Teflon sampling cups. Standard additions were carried out with Metrohm 765 Dosimats using a programmed dosing procedure (Noble et al., 2008).

For total dissolved cobalt analyses, samples were UV-irradiated for 1 h prior to analysis using a Metrohm 705 UV digester to degrade the organic ligands that bind cobalt and allow binding by the added electroactive cobalt ligand, dimethylglyoxime (DMG) (Saito and Moffett, 2001). Samples were analyzed in 8.5 mL aliquots with the addition of 30 μL recrystalized DMG (0.1 mol L^−1^ in methanol), 1.5 mL purified sodium nitrite (1.5 mol L^−1^ in Milli-Q water), and 50 μL purified N- (2-hydroxyethyl)piperazine-N-(3-propanesulfonic acid) (EPPS) buffer (0.5 mol L^−1^ in Milli-Q water). Reagent purification protocols were identical to those previously published (Saito and Moffett, 2001). Analysis began with a 180 s purge with 99.999% N_2_. Each sample was conditioned at −0.6 V for 90 s at a stir-rate of 2500 rpm followed by a 10 s equilibration step and a linear sweep from −0.6 V to −1.4 V at a rate of 10 V s^−1^. Cobalt concentrations were determined by the standard additions technique, with initial concentrations measured in triplicate followed by four 25 pmol L^−1^ cobalt additions. The analytical blank was determined by analyzing seawater that had been UV-irradiated for 1 h, equilibrated overnight with prepared Chelex 100 resin beads (Bio-Rad), and UV-irradiated a second time to degrade any leached synthetic ligands. Reagent blanks (nitrite, DMG, EPPS) were subtracted from the initial sample concentration, and blank analyses were made continually throughout the analysis of the sample set. The averaged blank concentration was 3 pmol L^−1^ ± 0.5 pmol L^−1^ (*n* = 7).

For labile cobalt analyses, 8.5 mL of sample were pipetted into acid-washed teflon vials that were preconditioned with a small aliquot of sample water. Thirty microliter of DMG were added to each vial and allowed to equilibrate overnight in the dark prior to analysis (Saito et al., 2004). Analyses were then performed as described for total concentrations with the addition of the remaining two reagents and use of the standard addition technique. Previously, we determined that natural cobalt ligands in seawater have a conditional stability constant of >10^16.8^ (Saito et al., 2005). This suggests that the cobalt is bound very tightly to the cobalt ligands. Thus, we define labile cobalt as the fraction of total dissolved cobalt that is either bound to weak organic and inorganic ligands in seawater or present as free Co^2+^, and is then exchangeable with the complexing agent (DMG) used for analysis (Saito et al., 2004, 2005). The difference between the total dissolved cobalt and the labile cobalt can then be used as an estimation of the strong cobalt ligand concentration.

### Total dissolved and total dissolvable iron and manganese analyses

Total dissolved and total dissolvable iron and manganese were measured using inductively coupled plasma mass spectrometry (ICP-MS), as described in detail by Saito and Schneider (2006). Total dissolved samples were filtered as described above, and total dissolvable samples were collected unfiltered. Briefly, 13.0 mL aliquots of acidified seawater were weighed into acid-leached polypropylene centrifuge tubes, to which an ^57^Fe solution was added for isotope dilution analysis and allowed to equilibrate overnight. Following equilibration, concentrated ammonium hydroxide (Seastar) was added to induce Mg(OH)_2_ and trace metal co-precipitation into a pellet. This was accomplished by allowing a precipitate to form for 3 min, followed by centrifugation for 3 min at 3000 rpm (1460 × g) using an Eppendorf Centrifuge 5810R. The supernatant was decanted, the sample was centrifuged a second time to further remove residual seawater, and the pellet was re-dissolved in 5% nitric acid (Seastar) made with 1 ppb indium. The 5% nitric acid resuspension solution was used to estimate the blank, and signal suppression due to matrix effects was accounted for by using a ratio of the indium in the blank to the indium in the resuspended sample solution. SaFe intercalibration standards were run concurrently with these samples and showed good agreement with current consensus values for Fe (D2: 0.923 ± 0.013n = 2, compared to consensus values of 0.933; S1: 0.108 ± 0.022n = 2, consensus value 0.093).

### Particulate metal analyses

For particulate metal analysis, filters were kept frozen at −20°C until analysis. Each filter was transferred to its own teflon digestion vial to which 4 mL of 50% nitric acid with 1 ppb indium was added as an internal standard. The filters and acid were then refluxed at 100–110°C for 6 h. Following digestion, the tubes were allowed to cool, and the remaining filter was removed from the digestion vials with trace metal clean plastic forceps. The samples were then diluted 1:10 with Milli-Q water to a 5% Nitric acid concentration. The dilutions were analyzed by ICP-MS with a 1–4 ppb metal reference standard curve (1, 2, 3, 4 ppb) to determine instrument sensitivity. Two processed filter blanks were run as well, where a cleaned, unused filter was digested following the above protocol. The metal values from this filter blank were subtracted from the sample blanks, corrected by the indium in each. Sample recovery and volume corrections post-indium addition were also corrected for by multiplying the metal counts per second (cps) by the ratio of indium cps estimated in the digest solution to the indium in the sample. The estimated indium cps in the 50% nitric digest solution (*In*_digestion_) was calculated by analyzing a 1:10 dilution of the 50% nitric digest solution to achieve a 5% nitric acid solution matrix match, and multiplying that value by 10. The equation used for calculations described above is:

$$M_{\text{particulate}}=\left[\frac{M_{\text{sample}}}{In_{\text{sample}}}-\frac{M_{\text{blank}}}{In_{\text{blank}}}\right]\times \frac{In_{\text{digestion}}}{M_{\text{slope}}}\times \frac{V_{\text{digested}}}{V_{\text{filtered}}}$$

where the *V*_filtered_ is the volume estimated to have passed through the filter (6 L), *V*_digested_ is the volume used to digest the sample (4.0 mL), *M*_sample_ is the metal of interest measured in the sample, *M*_blank_ is the metal of interest measured in the blank, *M*_slope_ is the slope of the metal of interest in cps ppb^−1^, *In*_*sample*_ is the indium measured in the sample in cps, *In*_blank_ is the indium measured in the blank in cps, and the concentration of the metal of interest (*M*_particulate_) is in ppb (μg L^−1^). The filter blank associated with particulate phosphorus was occasionally as much as 50% of the sample signal, reducing confidence in the quantitative values of particulate phosphorus, though it is unclear why the filter blank for phosphorus was high. All other analyses had filter blanks that were less than 6% of the signal, with the exception of cobalt, which occasionally was as high as 30%. This may be expected for cobalt, however, as suspended particulate cobalt concentrations are generally quite low (<10 pM).

### Sea ice core sampling and analysis

Sea ice cores were sampled with a 9 cm Kovac corer with a stainless steel blade and non-metallic, polyurethane body. Cores were sampled upstream of the expedition generator and the bottom layers were sampled into clean plasticware containers immediately upon retrieval from the ice. Samples were frozen immediately in liquid nitrogen dry shippers and transferred to a −80°C freezer until analysis. Digestion of sea ice material began by weighing an aliquot of sea ice, thawing it at room temperature with chilled oligotrophic trace metal clean seawater mixed 50:50 with the ice sample to avoid osmotic breakage of cellular material, and mixing. Triplicate subaliquots were collected and pelleted by centrifugation. The pellets were digested with 50% nitric acid 1ppb Indium solution at ~95°C for 3 h in sealed Teflon vials and left to cool overnight. The digests were then diluted to 5% nitric acid concentration and analyzed by Thermo Element 2 ICP-MS, using the same settings used for the particulate seawater analyses.

### Nutrient analyses

Nutrient analyses were processed at Oregon State University, using identical methods to those described in Noble et al. (2012). Briefly, nutrient samples were filtered and frozen in acid-washed 60 mL high-density polyethylene bottles until analysis. Samples were analyzed using an Alpkem autosampler. Technicon AutoAnalyzer II™ components were used to measure phosphate and the method was a modification of the molybdenum blue procedure of Bernhardt and Wilhelms (1967).

## Results

In order to put this dataset into a broader context, we compared it to observations of dissolved metals in the Ross Sea from two seasonal expeditions in 2005 and 2006 that comprised the Controls on Ross Sea Algal Community Structure (CORSACS) program (Saito et al., 2010; Sedwick et al., 2011). CORSACS-I took place during the austral summer of 2005–2006 (December 27, 2005–January 22, 2006), and CORSACS-II took place during the austral spring of 2006 (November 8–December 3, 2006).

### Total dissolved and labile cobalt distributions

**Figure 2:**
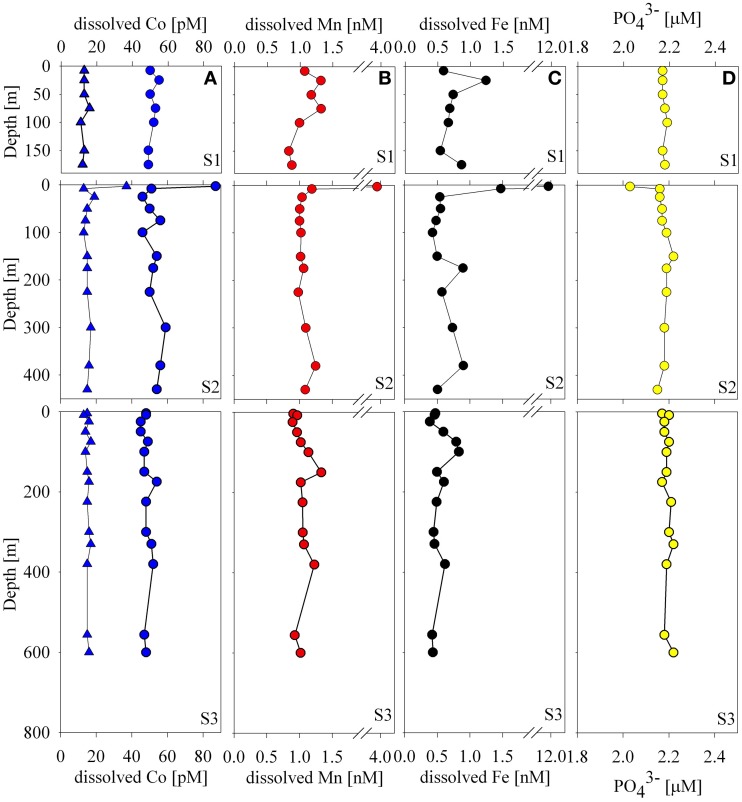
**Water column dissolved distributions of cobalt (A), manganese (B), iron, (C), and phosphate (D) at Stations 1–3.** Concentrations of total dissolved cobalt, labile cobalt, and phosphate were relatively uniform with the exception of the 4 m sample at Station 2 that showed elevated metals and slightly depleted dissolved phosphate. Iron and manganese trends were similar and varied within a relatively small range of concentrations with the exception of the surface sample at Station 2.

We observed two overriding features in the cobalt dataset that were consistent with our previous observations in the Ross Sea: (1) non-saturating concentrations of cobalt-binding ligands throughout the water column, and (2) no evidence of cobalt scavenging with depth, which is typically seen for this hybrid-type element in other oceanic regimes (Martin et al., 1989; Saito et al., 2004, 2010; Noble et al., 2008, 2012; Bown et al., 2011; Pohl et al., 2011; Biller and Bruland, 2012; Figure 2). As we have previously argued, the presence of labile cobalt throughout the water column may be attributable to the absence of marine cyanobacteria (Saito et al., 2010), which possess the biosynthetic pathway for synthesizing the important cobalt-containing vitamin B_12_ (Palenik et al., 2003; Rodionov et al., 2003; Sañudo-Wilhelmy et al., 2006, 2012), and are a potential source of strong cobalt binding ligands (Saito et al., 2005). The absence of these organisms likely leads to less complexation of cobalt in these cold waters (Saito et al., 2010). Although biochemical studies have discovered the presence of native heterotrophic bacteria that can synthesize B_12_ (Bertrand et al., 2011), their B_12_ production rates are likely low, indicated by observations of B_12_ and iron colimitation in the Ross Sea and Southern Ocean (Panzeca et al., 2006; Bertrand et al., 2007), where the observed B_12_ stimulatory response is inversely related to bacterial abundance (Bertrand et al., 2011).

The concentrations of total dissolved and labile cobalt were uniform with depth with the exception of one surface sample at Station 2 (Figures 1, 2). Manganese-oxidizing bacteria are known to co-oxidize cobalt during manganese oxidation (Lee and Tebo, 1994; Moffett and Ho, 1996; Tebo et al., 2004) and this process is thought to be a major removal pathway for cobalt in the water column. While considerable work has focused on understanding microbial manganese-oxide production in the water column of the marine environment (Tebo and Emerson, 1986; Tebo, 1991), questions still remain regarding the relative rates in the open ocean oxic water column. The absence of observable cobalt scavenging in the water column is consistent with slow and/or low manganese-oxide production coupled with mixing across the shelf (Saito et al., 2010, see section Particulate and Dissolved Cobalt and Manganese Distributions: Presence of Labile Manganese Particles and Fast Mixing).

### Total dissolved manganese and iron distributions

Total dissolved manganese concentrations ranged between 0.83 and 1.33 nM with the exception of the shallowest sample at Station 2 (3.88 nM, Figure 2). These concentrations are generally higher than deep water concentrations in the open ocean, which tend to fall between 0.1 and 0.3 nM (Martin and Knauer, 1985; Landing and Bruland, 1987) and higher than surface and deep waters found in the Atlantic sector of the Southern Ocean (~0.1–0.5 nM; Middag et al., 2011) with the exception of hydrothermal signals observed along the Mid-Atlantic Ridge where concentrations have been found to exceed 1nM (Middag et al., 2011; Saito et al., 2013). The concentrations found in this study are lower than many surface manganese concentrations in the tropical/subtropical ocean, which often display surface maxima due to aeolian input and photoreduction (Sunda and Huntsman, 1988). This seems reasonable given the decreased sources from the atmosphere to this region, and the fact that the waters are covered by sea ice for the majority of the year.

Total dissolved iron distributions were similar to those of manganese, though generally lower in concentration with a few exceptions. Iron concentrations ranged between 0.4 and 0.9 nM with the exception of 3 shallow samples (Station 1, 25 m, 1.2 nM; Station 2, 4 m, 11.9 nM; Station 2, 8 m, 1.5 nM; Figure 2). These concentrations were generally elevated above previously observed iron concentrations in the Ross Sea during both CORSACS expeditions (Sedwick et al., 2011), but were consistent with previous studies in the Ross Sea during early (October–November 1996; Coale et al., 2005) and late spring (November–December 1994; Sedwick et al., 2000).

### Particulate metal distributions

**Figure 3:**
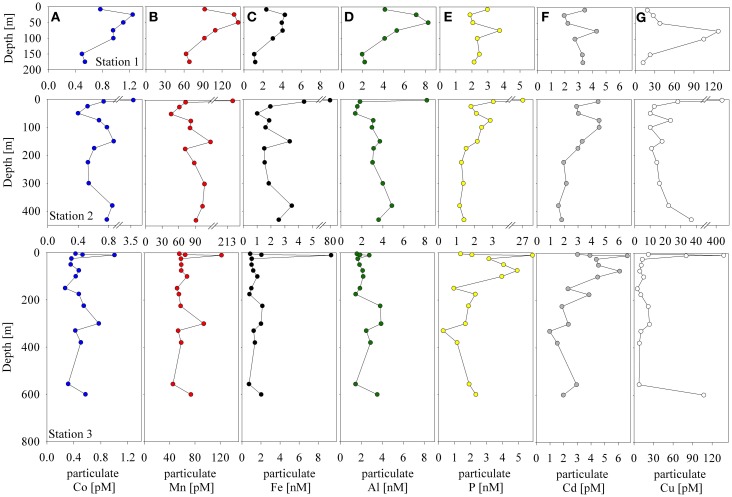
**Particulate cobalt (A), manganese (B), iron (C), aluminum (D), phosphorus (E), cadmium (F), and copper (G) distributions at Stations 1–3**.

**Figure 4:**
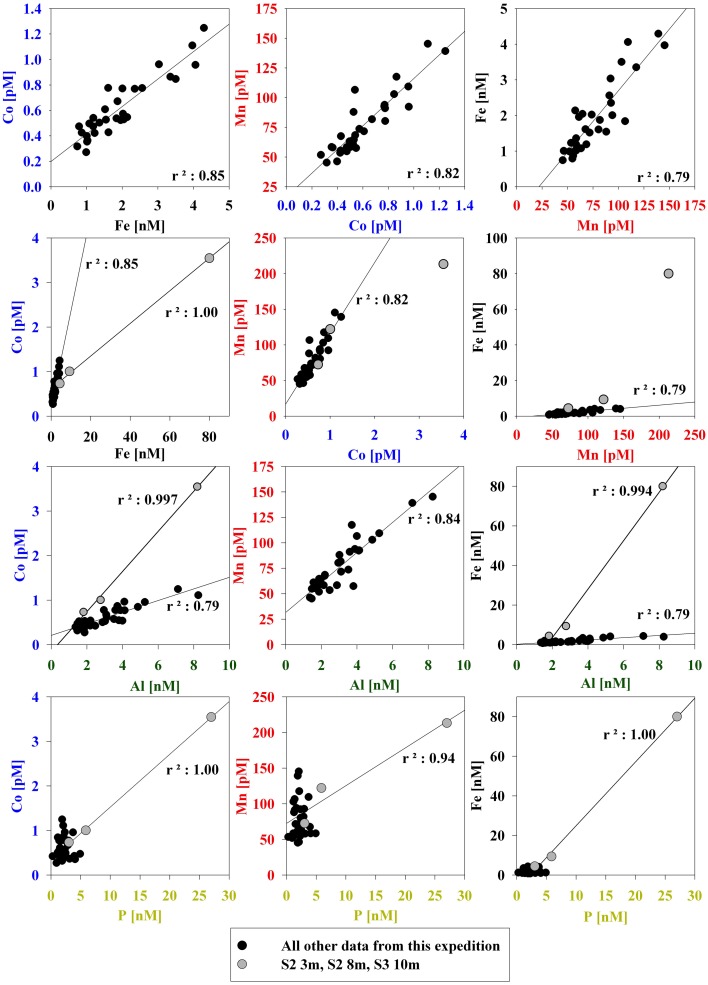
**Correlations between particulate bioactive trace metals for all three stations sampled.** Surface samples where biological activity was evidently high are shown in red (Station 2 = 3m, Station 2 = 8m, Station 3 = 10m).

**Figure 5:**
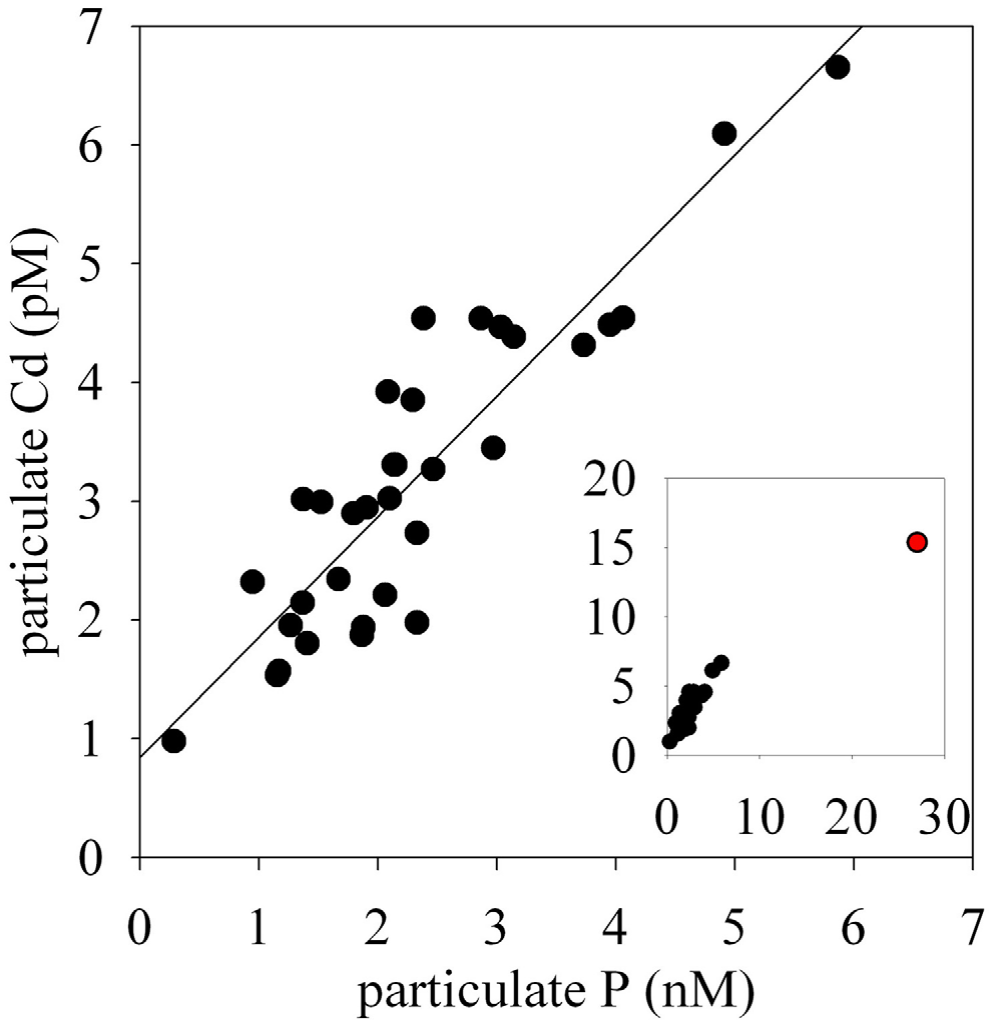
**A linear relationship between particulate Cd and particulate P**.

The particulate concentrations of cobalt, iron, and manganese had very similar distributions and showed more structure in the vertical profiles than was observed for the dissolved phase (Figure 3). When the particulate concentrations for all three stations were compared in paired scatter plots, strong linear relationships were evident between all pairings of metals, suggesting that their particulate distributions beneath the sea ice sheet were related by source or process (Figure 4). Strong linear relationships were also observed between aluminum and the hybrid-type metals. Samples that fell outside the general trend, shown in red, represent samples from the upper 10 m of the water column where the influence of biological processes was evident. These data showed linear relationships between Co, Fe, and P, though the relationship was driven by only three data points. Particulate cadmium, and copper were also measured (Figure 3), and a correlation between Cd and P, as has been previously characterized (Boyle et al., 1976; Cullen et al., 2003; Cullen, 2006; Hendry et al., 2008, 2010; Figure 5) was observed for all data with the exception of the shallowest sample at Station 2.

### Basal sea ice particulate metal abundances and chlorophyll a

**Figure 6:**
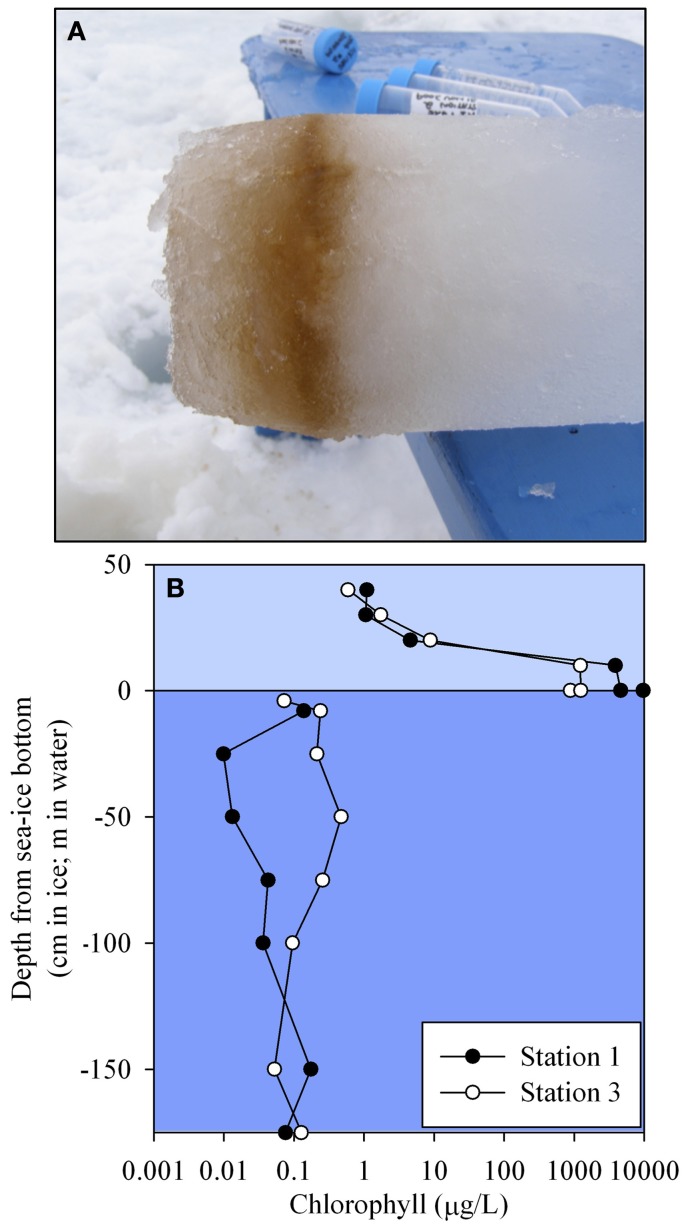
**(A)** Representative photo of a typical ice core bottom sampled during this field season, showing the thick basal layer of biomass. **(B)** Sea ice and water column chlorophyll *a* profiles for Stations 1 and 3.

Ice cores were collected at each station and the bottom of each core was analyzed for total metal and chlorophyll concentrations. The basal layer was visibly thick with biomass (Figure 6A) and contained extremely elevated concentrations of metals (Table 1). Concentrations are reported per liter of melted sea ice and reached concentrations that were more than 2 orders of magnitude higher than the extremely elevated shallow sample from Station 2 (9 μM compared to 80 nM particulate iron). With the exception of the core bottom at Station 2, the ratios of iron to aluminum in the ice were close to the values observed in the sediment (Table 1), suggesting that the material accumulating on the underside of the sea ice was likely derived from the sediment and/or was a major driver of the sediment composition. The elevated value found at Station 2 (9.7) is suggestive of iron being preferentially retained in this basal layer relative to aluminum and this is discussed in a later section as a potential capacitor for iron during the winter months (See section Basal Sea Ice Biological Communities as a Capacitor for Iron). The manganese to aluminum ratios were slightly elevated above the sediment values (Table 1), but much lower than the average water column particulate ratios. This suggests that an accumulation of manganese over aluminum relative to the sea ice was occurring in the water column (See section Particulate and Dissolved Cobalt and Manganese Distributions: Presence of Labile Manganese Particles and Fast Mixing). It is also likely that a fraction of the total metal in the ice core was from a leachable particulate fraction. This has been observed previously in the Bering Sea, where total dissolvable iron was found in ice cores at values ranging from 111 nM to 75 μM, compared to the dissolvable fraction, which ranged from 2.9 to 376 nM (Aguilar-Islas et al., 2008). The leachable material in that study was found to be sediment derived. Though the dissolved fraction was not assessed for the ice cores in this study, leachable particulate metals here were most likely a combination of sediment material and biomass given the shallow water column depth, proximity to islands, and the visual observation of biomass in the basal layer (Figure 6A).

**Table 1:** Average metal abundances and ratios to Aluminum from this study and comparison to nearby sediment (^*^Ravanelli et al., 1997 and Ianni et al., 2010) and upper crust (^+^Taylor and McLennan, 1985).

**Sample**	**Mn [nM]**	**Fe [nM]**	**Co [pM]**	**Fe/Al**	**Mn/Al**	**Co/Al**
Near bottom ice core	320	865	5883	0.4	15.1	2.916
Ice core bottom Stn.1	209	6132	5790	0.39	13.3	0.372
Ice core bottom Stn. 2	135	9318	4270	0.77	11.3	0.36
Ice core bottom Stn. 3	777	3470	3670	0.44	10.3	0.898
Surface sample Stn. 2 pM	0.21	80	3.5	9.7	39.6	0.404
Surface sample Stn. 2 dM	3.9	12	87			
Average water column pM	0.08	1.9	0.6	0.59	25.9	0.197
Average water column dM	1.1	0.6	50			
Sediment^*^				0.48	8.6	0.17
Crust^+^				0.21	3.7	0.057

Chlorophyll *a* concentrations revealed intense biological activity on the underside of the sea ice with values in excess of 9500 μg/L compared to the orders of magnitude lower concentrations found in the more isolated sea ice layers (1 μg/L) and water column (0.1 μg/L, Figure 6B). These concentrations were consistent with previous high reports in this region (Palmisano and Sullivan, 1983; Grossi et al., 1987; Garrison et al., 2003), although higher than sea ice chlorophyll *a* reviewed throughout Antarctica over the last 25 years (405 μg/L, Meiners et al., 2012). Interestingly, a similar sea ice study by Grotti et al. (2005), found chlorophyll *a* concentrations in the bottom 10 cm of an ice core from nearby Terra Nova Bay in November 2001 of 34 μg/L, yet found iron concentrations in that portion of the core (1015 nM) to be similar to those found in the “near bottom” core sample from this study (865 nM, Table 1). Seasonal sea ice sampling from that study observed a coupling between chlorophyll *a* and particulate metal concentrations on the underside of the sea ice, which they attributed to incorporation into particulate organic matter (Grotti et al., 2005).

### Comparison of acid leachable particulate iron to particulate iron

In addition to filtered and particulate analyses described above, unfiltered samples were also collected and acidified for total dissolvable iron and manganese analyses. The interpretation of the unfiltered fraction is a subject of study because different analytical techniques may detect different fractions of the total metal (Bowie et al., 2004). Previous studies have used the difference between analyses of unfiltered samples (total dissolvable) and filtered samples (total dissolved) to infer the acid leachable particulate iron concentration (ALP Fe hereon; Chever et al., 2010; Sedwick et al., 2011). These studies have generally employed flow injection analysis techniques, which likely do not detect refractory iron that may pass through the instrument. A comparison study between different flow injection analysis techniques has observed slight differences likely due to the detection of different physiochemical fractions of iron (Bowie et al., 2004). Other work has suggested that analysis of the unfiltered fraction using ICP-MS techniques may more closely represent the total iron than the total dissolvable iron, as particles may be precipitated during the co-precipitation preconcentration step, and then subsequently ionized if taken up into the instrument during analysis (Boyle et al., 2005). This may lead to slight differences in an ALP Fe inferred from ICP-MS analyses relative to those inferred from flow injection analyses. In this study, we analyzed unfiltered, filtered, and particulate filter samples by ICP-MS. The unfiltered and filtered samples are referred to as total dissolvable and total dissolved respectively, and were used to estimate the ALP Fe as described above, similar to those discussed in Sedwick et al. (2011).

**Figure 7:**
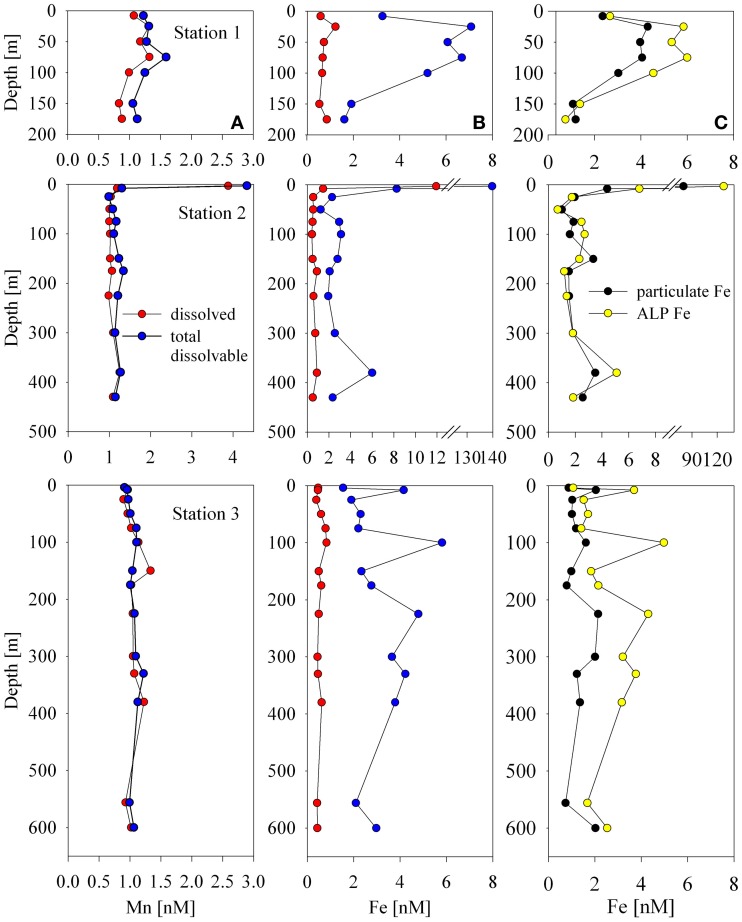
**A comparison of total dissolvable to dissolved manganese (panel A profiles) and total dissolvable to dissolved iron (panel B profiles).** The acid leachable particulate concentration is determined by the difference between these two concentrations. This demonstrates that the acid leachable particulate iron should be resolvable using this method, and that the acid leachable particulate manganese fraction appears to be very small. Panel **(C)** profiles compare the calculated acid leachable particulate iron (ALP Fe) to particulate iron measurements.

The unfiltered samples were analyzed after storage and leaching under acidic conditions for at least 3 months using the same analytical protocol described for the total dissolved analyses (see methods section). Figure 7 shows the results of the total dissolvable and total dissolved analyses for both iron (middle panel profiles) and manganese (left panel profiles). A large difference between the total dissolvable and total dissolved iron indicates that the ALP Fe metal should be resolvable. In contrast, the difference between the total dissolvable manganese and total dissolved manganese was small, suggesting that the ALP Mn was a small fraction of the total, consistent with the measured particulate manganese being ~20 times less than the total dissolved manganese (Figure 3).

When ALP Fe is compared to particulate Fe, a couple of observations can be made (Figure 7, right panel profiles). The broad trends observed in the particulate Fe profiles were also observed in the ALP Fe profiles, and the ALP Fe and particulate Fe below the surface at Station 2 agreed well, however; the particulate Fe concentrations were generally lower than the ALP Fe, especially at Stations 1 and 3 (Figure 7). This was unexpected since the particulate samples were digested with a strong acid (50% HNO_3_) at high temperature, but the ALP Fe values were derived from analyses of samples that were weakly acidified (pH 1.7 with HCl) at room temperature. Both the filtered and unfiltered samples were acidified with the same batch of acid, the bottles were treated identically during cleaning, and were analyzed using the same technique, so contamination is unlikely. There are three possible explanations for these results. First, the higher values derived from the unfiltered samples may be due to the ionization of refractory iron that may have precipitated during the Mg(OH)_2_ coprecipitation preconcentrating step and been carried into the instrument during analysis as has been suggested by Boyle et al. (2005). Second, a combined acid mixture that includes HF is often used to digest all recalcitrant lithogenic material for total particulate analyses (Sherrell and Boyle, 1992; Cullen et al., 2003; Chever et al., 2010; Lannuzel et al., 2010, 2011), which might account for a lower particulate metal detected by the digestion used for this dataset which did not include HF. This seems unlikely though, because the Fe/Al ratios in the particulate samples were very similar to that of nearby sediment that had been digested with nitric acid and HF (Ianni et al., 2010). Even if all lithogenic material was not fully digested by the 50% nitric acid leach, our objectives were to analyze labile manganese oxides and cobalt associated with them for which nitric acid was sufficient. Third, a few of the total dissolvable samples were re-analyzed a month later, and the concentrations measured were slightly higher, implying that iron was continually being released from particulate matter in the sample bottles with time. These results suggest that future efforts are needed to characterize the differences between ALP Fe and pFe, as well as the influence of different acid leaches on the dissolution of a range of particulate phases, and to document systematic differences associated with the instrumental techniques used for analysis (Lam and Ohnemus, pers. commun.).

## Discussion

### A lingering biological signal in dissolved Co:PO^3−^_4_ ratios

**Figure 8:**
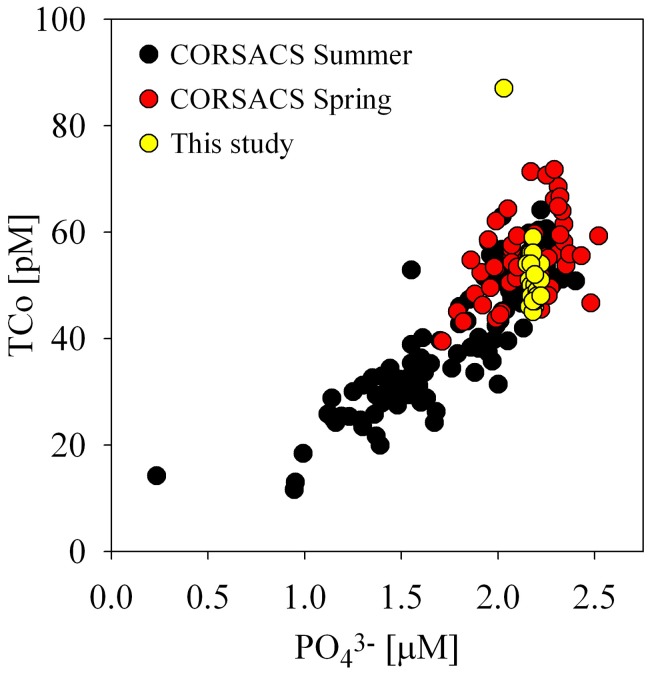
**A comparison of dissolved cobalt and phosphate relationships under the sea ice (this study) and in the Ross Sea Polynya (CORSACS spring and summer, Saito et al., 2010).** The Co:PO^3−^_4_ ratios observed under the sea ice were consistent with previous early season polynya Co:PO^3−^_4_ ratios.

During both CORSACS studies, total dissolved cobalt correlated strongly with soluble reactive phosphate throughout the water column, implying that biological uptake and remineralization were the primary influences on cobalt distributions during the spring and summer (Saito et al., 2010; Figure 8). The small range of water column concentrations of both cobalt and phosphate observed during this study did not appear to have a strong linear relationship, however; when compared to our data from CORSACS, the Co:PO^3−^_4_ ratio range for the under sea ice dataset fell along the previously observed relationship, consistent with early season input and winter mixing homogenization of dissolved distributions (Figure 8). The coherence of the McMurdo Sound data with the upper values of the CORSACS cobalt data suggests that the biologically driven Co:PO^3−^_4_ ratio observed in the Ross Sea polynya (Saito et al., 2010) persisted in the water column even through the end of the sea-ice season, and that processes did not deplete either nutrient relative to mixing rates (Figure 8). While the lack of significant under ice vertical structure is perhaps not surprising given the lowered water column phytoplankton productivity (Figure 6B), it is interesting that evidence of scavenging was not detectable in the water column after the long winter season, and this is discussed in further detail in the following section (Particulate and Dissolved Cobalt and Manganese Distributions: Presence of Labile Manganese Particles and Fast Mixing).

### Particulate and dissolved cobalt and manganese distributions: presence of labile manganese particles and fast mixing

The lack of a scavenged profile for cobalt with depth raises questions about the absence of removal processes and speed of mixing processes beneath the sea ice. Previously, we observed a lack of cobalt scavenging in full depth water column profiles from the Ross Sea, and hypothesized that scavenging by manganese-oxidizing bacteria was slow, due to reduced populations or activities of these bacteria in the cold Antarctic waters, and/or that deep seasonal mixing was occurring on timescales fast enough to erase any detectable influence of removal processes on the dissolved cobalt distributions (Saito et al., 2010). Particulate metals were not measured during that study to look for evidence of manganese oxide particles, and the combination of both dissolved and particulate cobalt and manganese data here help discern which mechanism has a stronger impact on scavenging in the Ross Sea.

In the particulate data, coincident maxima of manganese and cobalt were suggestive of manganese oxidation and co-oxidation of cobalt by manganese-oxidizing bacteria either in the water column or in resuspended sedimentary material (Figure 3). At Station 2, an increase in particulate manganese and a concomitant but smaller increase in particulate cobalt were observed between 75 and 175 m depth (Figure 3). There was also a maximum of particulate manganese at 300 m in Station 3 (Figure 3). Particulate cobalt and manganese correlated exceptionally well with only one exception in the surface sample from Station 2 (Figure 4). This also supports the proposed coupling of the particulate phases of these metals through the bacterial manganese oxidation pathway (Tebo et al., 1984). It is important to note that our study can examine for the presence of manganese particles, but does not measure water column manganese and cobalt oxidation rates directly as previous studies have (Moffett and Ho, 1996). Hence, we cannot discern if any manganese particles present were formed in the water column or advected from sedimentary material, however; the particulate Mn/Al ratios observed in the water column were significantly elevated relative to both a local sediment core study and crustal values, suggesting that the excess particulate manganese is a result of manganese oxide production occurring in the water column. In contrast to observations of elevated particulate concentrations coincident with dissolved minima that have been observed in the Equatorial Pacific (Cowen and Bruland, 1985, Saito, unpublished data); if manganese oxides from manganese-oxidizing bacteria are present in this polar environment, the evidence was observable in the particulate profiles only, and not in the dissolved. This suggests that water column mixing was faster than the influence of biotic cobalt and manganese oxidation by manganese-oxidizing bacteria. Additionally, tight coupling of particulate cobalt, iron, manganese, and aluminum was observed. This implies that while manganese-oxide production may be occurring in the water column, the particulate signals are more strongly driven by tandem cycling of these metals with sediment resuspension, as discussed in a later section (Consideration of Island Effects, Terrestrial Input, and/or Sedimentary Influences). Sediment in McMurdo Sound is often dominated by biogenic rainout from summer blooms, consisting of 40–50% organic matter and/or biogenic silica (Ravanelli et al., 1997; Atkins and Dunbar, 2009; Ianni et al., 2010). The Ross Sea may be too shallow to observe removal by manganese-oxidizing bacteria, and active sediment resuspension effectively masks this water column scavenging process.

**Figure 9:**
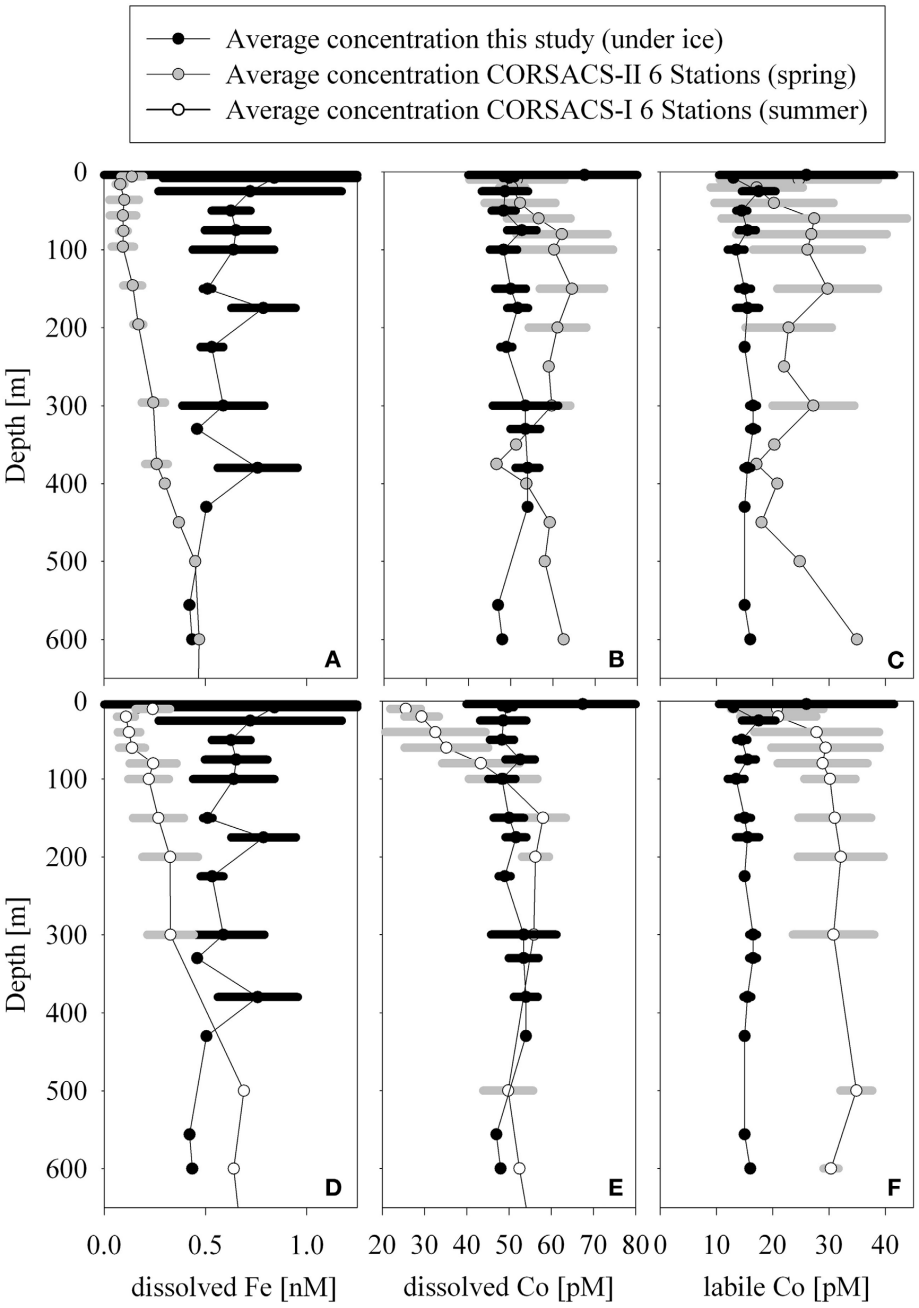
**Averaged profiles of dissolved iron (A,D), dissolved cobalt (B,E), and labile cobalt (C,F) from this study (all panels, Stations 1–3), the spring CORSACS study (A–C, Stations NX12–15, NX17, NX20; data from Saito et al., 2010; Sedwick et al., 2011), and the summer CORSACS study (D–F, Stations NX1–4, NX7, NX10; data from Saito et al., 2010; Sedwick et al., 2011).** Stations from the CORSACS study were chosen based on proximity of station location to this study. A similarity between the intermediate depth concentrations of total dissolved cobalt during this study and the summer CORSACS study was apparent. While total cobalt was drawn down in surface waters over the season, labile cobalt was higher in the spring than under the McMurdo sea ice, perhaps due to the introduction to deeper waters via remineralization in the spring and summer. The biotic drawdown of iron appeared to penetrate to deeper depths than observed for cobalt.

The concentration range of total dissolved cobalt observed in this dataset (50 ± 3.5 pM, *n* = 32) was similar to those previously observed below the euphotic zone during the summer in the Ross Sea (53 ± 4.5 pM *n* = 44, CORSACS-I study Saito et al., 2010; Figure 9). Biological drawdown in the summer decreases the surface concentrations of total dissolved cobalt in the Ross Sea relative to the concentrations observed during this study beneath the McMurdo Sound sea ice (Saito et al., 2010). When compared to the spring CORSACS-II cobalt distributions, however; total cobalt concentrations were higher in the Ross Sea than beneath the sea ice (Figure 9). Interestingly, the labile concentrations observed during both CORSACS expeditions were higher than those observed during this study (Figure 9). While loss of total and labile cobalt has been observed over time for samples collected within oxygen minimum zones (Noble et al., unpublished data), these waters are well-oxygenated, and repeat analyses performed on a subset of the CORSACS samples greater than 17 months after collection gave similar values to those obtained at sea for both the total and labile fractions. Thus, we do not believe that the difference in labile cobalt observed between the McMurdo samples (analyzed 2 months after sampling) and CORSACS samples (analyzed at sea) is due to loss of labile cobalt over time.

### Under ice dissolved iron concentrations and the winter reserve

The dissolved iron concentrations were relatively high for this region (0.4–0.9 nM), consistent with the notion of a winter reserve of high water column dissolved iron (Sedwick et al., 2000). The timing of the transition from winter reserve to iron-limiting conditions in the polynya are of increasing interest (Bertrand et al., 2011; Sedwick et al., 2011). During the spring CORSACS-II expedition, dissolved iron concentrations were significantly depleted to sometimes below 0.1 nM in surface waters due to biological uptake during the spring bloom (Figure 9), however; some deeper waters displayed elevated concentrations that were consistent with the concept of a “winter-reserve” hypothesized by that study (Sedwick et al., 2011). The upper water column average dissolved iron concentrations for both seasons of the CORSACS study are lower than those observed beneath the McMurdo Sound seasonal sea ice (Figure 9). This trend is the opposite of what was observed for cobalt, where the spring total dissolved and labile cobalt CORSACS-II concentrations were found to be higher than those observed beneath the McMurdo Sound seasonal sea ice. This could be due in part to a higher biological demand for iron relative to cobalt. If the waters sampled during this expedition were representative of winter distributions beneath the seasonal sea ice across the coastal regions of the Ross Sea, these finding are consistent with the hypothesis that well-mixed “winter-reserve” shelf water could supply iron to surface waters during the spring bloom. Interestingly, however, recent results demonstrate iron depletion (Sedwick et al., 2011) and resultant iron limitation (Bertrand et al., 2011) during the early polynya formation, implying that the dissolved winter reserve itself is not sufficient to support the intense annual spring bloom. Particulate sources may be particularly important in resolving this conundrum.

### Consideration of island effects, terrestrial input, and/or sedimentary influences

**Figure 10:**
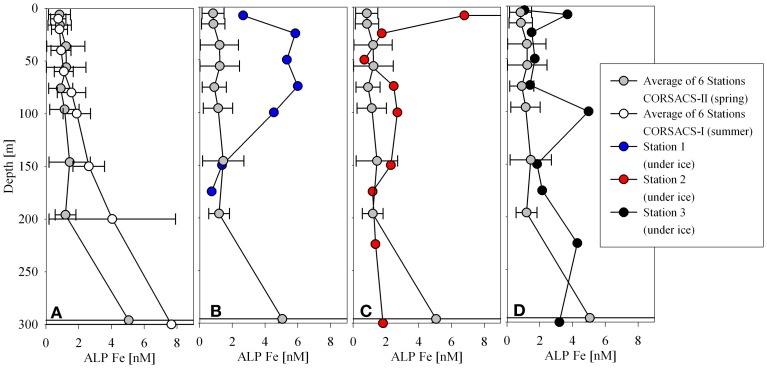
**A comparison of acid leachable particulate iron from the CORSACS expeditions (A, Sedwick et al., 2011) to acid leachable particulate iron profiles at Stations 1 (B), 2 (C), and 3 (D)**.

The proximity of the stations to the large, Ross Island and smaller Delbridge Islands (Figure 1) suggests that terrestrial inputs may contribute to the dissolved and particulate water column distributions. Evidence of sources from this region could be seen when the ALP Fe distributions from this study were compared to the ALP Fe distributions from both CORSACS studies (Sedwick et al., 2011; Figure 10). While the data from the CORSACS study are temporally different from the sampling efforts of this study, they represent a sample set that describes the distributions following a spring melt. Even with these comparison limitations, the trends suggest influence from terrestrial inputs.

The average concentrations of 6 stations from the spring (Stations NX12–15, 17, 20) and summer (Stations NX1–4, 7, 10) CORSACS cruises are shown relative to each of the stations from this study (Figure 10). The ALP Fe values from the CORSACS expeditions were lower in the upper 200 m than observed during our field season, and did not vary considerably from spring to summer. Much higher concentrations of ALP Fe were observed during this study at Station 1, located between the Delbridge Islands and Ross Island (Figure 1), and may be due to contributions from the sediments or aerosols given the proximity of this station to small islands with volcanic-rich soils. Aeolian sources to Southern McMurdo Sound primarily affect sediments in proximity (<10 km) to the McMurdo Ice Sheet over which strong northward winds carry volcanic-rich particles from exposed parts of Ross Island and the Antarctic continent (Atkins and Dunbar, 2009). Similarities between the composition of ice samples taken in proximity to Ross Island and that of sediment samples from within Southern McMurdo Sound led these workers to suggest that aeolian deposition does contribute to the sediment load, although the sampling sites described in that study are to the South and West of the this study, and our sampling sites may be further removed from aeolian sources. The elevated ALP Fe observed at Station 1 may also be reflective of the shallow water column depth. Some stations from the CORSACS expeditions showed significant evidence of sediment mobilization by elevated ALP Fe concentrations up to 68 nM at depth, but those stations had bottom depths as deep as 700 m and are not shown here (Sedwick et al., 2011). Station 1 had a water depth of approximately 190 m, and the elevated iron observed there is likely due to sediment resuspension. The particulate iron maximum at Station 1 was 4.5–6.0 nM ALP Fe, 3.0–4.3 nM particulate Fe, however; these signals were considerably smaller than the high concentration observed in the surface sample at Station 2 (128 nM ALP Fe, 80 nM particulate Fe) which is discussed in the following section (Basal Sea Ice Biological Communities as a Capacitor for Iron).

**Figure 11:**
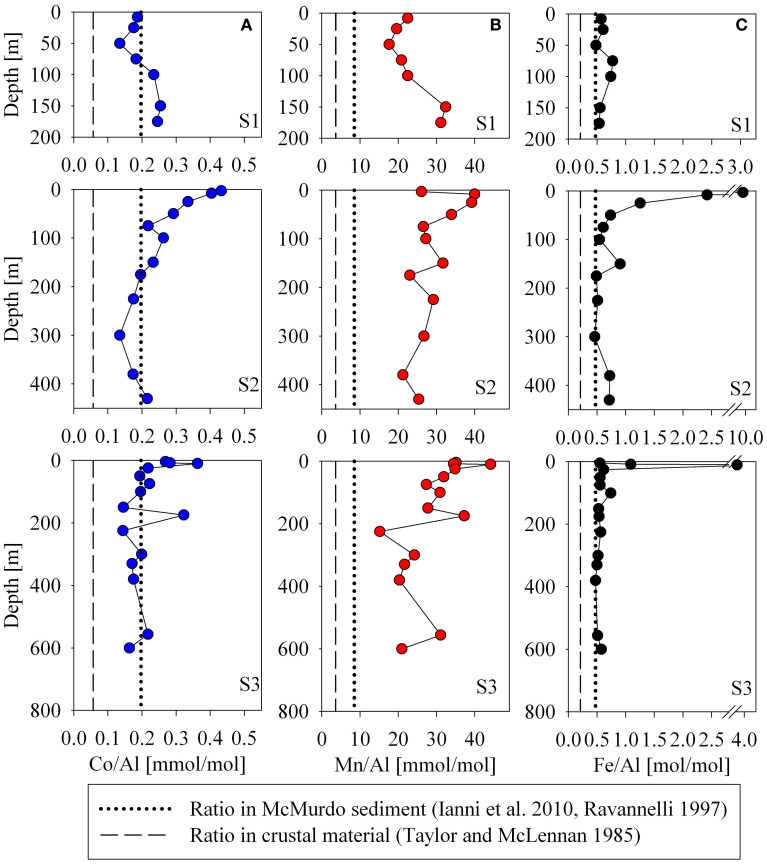
**Aluminum normalized particulate metal profiles for cobalt (A), manganese (B), and iron (C).** The Fe/Al ratios are similar the sediment composition value for two Ross Sea sediment core study sites (dotted lines, Ravanelli et al., 1997; Ianni et al., 2010), but both the sediment and water column ratios are higher than crustal ratios (dashed lines, Taylor and Taylor and McLennan, 1985), suggesting that the particulate influences observed here are more biogenic than lithogenic.

The influence of terrestrial/continental sources can be inferred by comparing ratios of each metal with aluminum in the particulate phase to nearby sediment and to crustal values. Using data from two Ross Sea sediment core studies for iron and manganese (Ianni et al., 2010) and for cobalt (Ravanelli et al., 1997), Figure 11 shows the relationships between the metal/Al ratios in the suspended particulate material, the crustal ratios (Taylor and McLennan, 1985), and that of the local sediment. Particulate and sediment core Co/Al, Fe/Al, and Mn/Al ratios were all higher than the respective metal/Al ratios of crustal material, indicative of biogenic content in the particles and sediment because Al is not appreciably accumulated in biogenic material relative to lithogenic material. The Fe/Al ratio found at most depths for all stations was close to that of the sediment. The particulate Mn/Al ratios, however, far exceeded that of the sediments (Mn/Al in the sediments: 8.5 compared to Mn/Al in the suspended particles: 25.9, Table 1), consistent with a biogenic component produced by manganese-oxidizing bacteria as discussed earlier (section Particulate and Dissolved Cobalt and Manganese Distributions: Presence of Labile Manganese Particles and Fast Mixing, Figure 11). The Co/Al sediment core ratio was determined after a cold, weak acid leach (0.3 N HCl at room temperature for 2 h, Ravanelli et al., 1997). As a result, the comparison of sediment to water column Co/Al ratios may differ from that of iron and manganese. This could bias the sediment Co/Al ratio toward values that are higher than reflective of the total sediment composition since a cold, weak acid leach could dissolved most of the particulate cobalt as it is not typically considered to be a silicate forming metal, but would not dissolve all the aluminum, which tends to be elevated in the more recalcitrant lithogenic fraction. Thus, it is possible that there may be cobalt oxides produced in the water column as a result of co-oxidation by manganese oxidizing bacteria, but similar to the manganese distributions, if this is the case, the evidence is not discernable in the dissolved phase.

Interestingly, there was no large bottom depth point source observed for either the particulate iron or ALP iron profiles (Figure 7). This may have been a result of winter mixing across much of the sound and/or the digestion used for these analyses. For total particulate digestions, hydrofluoric acid (HF) is often used. No HF was used for the particulate analyses in this study, and some lithogenic sources may be missed, however; the similarity observed between the Fe/Al ratio in most of the suspended particulate analyses and the Fe/Al ratio of the sediment core from the Ross Sea (Ianni et al., 2010) suggests that the suspended particulate material sampled may have been fully digested by the nitric acid treatment. The values that were used for the Fe/Al and Mn/Al sediment composition comparison were determined after a nitric acid and HF digestion (Ianni et al., 2010).

Lastly, ratios of Co/Al, Fe/Al, and Mn/Al were all in significant excess of the sediment and crustal ratios for the shallow samples at Station 2 (Figure 11), where elevated concentrations of dissolved and particulate Co, Fe, and Mn were observed coincident with a slight drawdown in dissolved PO^3−^_4_ (Figures 2, 3). This suggests that the elevated metal signal observed there was due primarily to biogenic sources of metals rather than from lithogenic material, supporting a potential biological source mechanism to introduce high concentrations of labile metals to shallow waters of the Ross Sea during the spring melt, discussed in the following section (Basal Sea Ice Biological Communities as a Capacitor for Iron).

### Basal sea ice biological communities as a capacitor for iron

A striking feature in this dataset was the elevated dissolved and particulate concentrations found in the shallowest sample of Station 2 for all metals measured except cadmium (Figures 2, 3). This observation implies that the sea ice in coastal areas around the continent could be an important source of these metals to the water column. Examination of the iron content in the bottom layers of the sea ice confirms an extraordinarily high iron load present in these biologically rich layers (Table 1), with concentrations of up to 9 μM. Moreover, our combined datasets (the coordinated water column, sea ice basal layer, and particulate metal sampling) from a transect away from the Ross and Delbridge Islands together provide confirmation of island/sedimentary sources as well as a trend between biomass and basal particulate iron concentration. In particular, intense biological production was observed in the bottom 2–3 inches of the cores as thick brown biomass at all three stations (Figure 6A) with increasing biomass closer to shore. The maximum chlorophyll *a* concentrations found in this bottom basal layer (9500 μg/L) were well above the values reported in a recent review of sea ice chlorophyll (Meiners et al., 2012), but are consistent with previous reports of high sea ice biomass in McMurdo Sound (Palmisano and Sullivan, 1983; Grossi et al., 1987; Garrison et al., 2003). The phytoplankton community was dominated by diatom species, as previously described for McMurdo Sound and of similar high biomass (Grossi et al., 1987; Garrison et al., 2003). The species assemblage and concurrent transcriptomic and proteomic analyses will be presented elsewhere (Saito et al., in preprepation).

Our transect showing a trend in increasing concentrations of both iron and chlorophyll with proximity to McMurdo Sound Islands (Ross and the Delbridge Islands) implies a connection to these sources. There is likely an annual cycle for iron accumulation within the bottom biologically rich layers of the sea ice near these island sources, where the phytoplankton communities are also reliant on these coastal sources to avoid the iron limitation that is dominant throughout the Southern Ocean. Early studies on sea-ice trace metals removed and discarded the bottom layer of sea-ice due to concern for contamination (Lannuzel et al., 2006, 2007; Aguilar-Islas et al., 2008), but more recent studies included these layers and found high particulate metals presumably believed to be associated with biogenic material (Lannuzel et al., 2011). Our observations during the two expeditions in 2009 found that the biologically rich 2–3 inch basal layers of the sea ice present in the early season were entirely sloughed off by February at similar McMurdo Sound sampling locations, indicating a seasonal cycle of accumulation and release from the sea-ice base, prior to the sea ice breakup. This biologically supported accumulation and sloughing-off process is likely an important mechanism for release of coastal particulate iron supply coastal Antarctic regions. In support of this biochemical concentrating mechanism at the sea ice-seawater interface, the shallow sample at Station 2 was also the only observation of detectable drawdown of water column PO^3−^_4_ (Figure 2). This notion builds upon the importance of particulate iron described by Lam and Bishop for the global oceans (2008), but adds a means of accumulating and releasing the iron at the base of the sea ice, promoting a potentially more intense and temporally constrained release. This basal sea ice capacitor-like storage capability for iron is similar to that described for coastal sediments (Chase et al., 2007), and hence makes for appropriate terminology here. Seasonal sea ice has been described for its role in accumulating metals via aeolian deposition and/or from entrainment of organic and inorganic terrestrial and near shore sedimentary material (Atkins and Dunbar, 2009; Sedwick et al., 2011), yet its role as a capacitor for accumulating iron and its potential influence on water column phytoplankton nutrition has only recently come to attention as being a potentially vital mechanism contributing to the spring melt iron supply (Saito et al., 2011, Polar Marine Sciences Conference; Noble et al., 2013, ASLO meeting).

It is interesting to place these findings in the context of this largely iron limited Ross Sea region. Recently, Sedwick et al. (2011) demonstrated that very low dissolved iron concentrations, sometimes below 0.1 nM (Figure 9), were present in the early spring/summer during polynya formation, resulting in iron limiting conditions in November/December Ross Sea incubation experiments (Bertrand et al., 2011). These findings suggest that there is a rapid transition between the “winter reserve” (Sedwick et al., 2011), observed here under the sea ice, and the upper water column iron depletion and iron limited conditions. The extremely high abundances of particulate metals within the sea ice community could help explain this rapid transition: our observations are consistent with nearby island sources and the associated gradient of increased particulate metals and chlorophyll biomass closer to the islands, as well as high overall biomass reported in the sea ice of McMurdo Sound. The enhancement of particulate metals in the water column near this sea ice biota community, particularly in proximity to coastal environments, also points to the basal sea ice layers serving as a potential reservoir of particulate metals that are released during bottom sloughing of sea ice that occurs in McMurdo Sound between December and January/February. McMurdo Sound has an anti-cyclonic circulation pattern with Ross Sea waters moving southward through the eastern basin and waters from under the Ross Ice Shelf moving northward along the western side of the sound (Barry and Dayton, 1988), consistent with observations of biogenic flux in the Sound (Dunbar et al., 1989). While much of the basal sea ice iron is likely lost to the sea floor with the biogenic flux, a component likely remains in the water column and if advected to the open water polynya region, it could contribute to promoting and sustaining the annual phytoplankton blooms that occur there. Moreover, enriched basal communities of sea ice are observed within the Ross Sea during early season ice-breaking transits (Noble and Saito, personal observation), and those communities could also contribute to a sloughing iron flux. Hence this sea-ice iron capacitor process could be important in coastal regions throughout Antarctica given recent evidence of high iron fluxes in other coastal regions such as the Amundsen Sea (Planquette et al., 2013). The regional influence of sea-ice basal communities for polynya blooms should be further examined through future sampling and regional modeling studies.

## Conclusions

Dissolved and particulate cobalt, iron, and manganese distributions in the water column beneath the seasonal sea ice reveal that winter concentrations of these important metal micronutrients were relatively high during the Spring of 2009. The proximity of the sampling stations to nearby islands, and the tight coupling of the particulate phases of all three metals suggests that the dissolved distributions may be strongly influenced by sediment resuspension. Similar Fe/Al ratios in the sediment and water column particles suggest that at least in these waters, the 50% nitric acid leach may have digested the majority of the particulate material, which may not be surprising given the expected strong organic component of sediments in this area. In contrast to during the summer season, biological activity appears to have minimal influence under the sea ice on the water column distributions of hybrid-type metals. In the shallowest samples, however, high concentrations of iron, cobalt, and manganese were observed, coincident with a slight drawdown of PO^3−^_4_ and thick brown biomass on the underside of the sea ice. The presence of manganese-containing particles was observed in the particulate profiles, but without a concomitant decrease in dissolved manganese and cobalt, as would be expected with precipitation by manganese-oxidizing bacteria, suggesting that the rate of water column mixing was faster than the biotic oxidation of dissolved cobalt and manganese. These results agree with our previous hypothesis that micronutrient uptake drives nutrient-like distributions of cobalt observed in the summer (Saito et al., 2010), and that the conservative distributions observed in the winter are a result of fast water column mixing, and decreased concentrations or activities of manganese-oxidizing bacteria. These results also support previous hypotheses that elevated “winter-reserve” concentrations of metals exist beneath the sea ice (Sedwick et al., 2011). Lastly, the coordinated water column, sea ice basal layer, and particulate metal sampling from this study provides evidence of under sea ice accumulation of metals by the resident biota and the potential role of the sea ice basal layer as a capacitor for accumulating iron during the early spring. This sea ice iron source could play a role in water column phytoplankton nutrition in McMurdo Sound and the Ross Sea.

### Conflict of interest statement

The authors declare that the research was conducted in the absence of any commercial or financial relationships that could be construed as a potential conflict of interest.
